# The Pattern of Fatty Acids Displaced by EPA and DHA Following 12 Months Supplementation Varies between Blood Cell and Plasma Fractions

**DOI:** 10.3390/nu7085285

**Published:** 2015-08-03

**Authors:** Celia G. Walker, Annette L. West, Lucy M. Browning, Jackie Madden, Joanna M. Gambell, Susan A. Jebb, Philip C. Calder

**Affiliations:** 1 MRC Human Nutrition Research, Elsie Widdowson Laboratory, Fulbourn Road, Cambridge CB1 9NL, UK; E-Mails: celia.walker@mrc-hnr.cam.ac.uk (C.G.W.); lmbrowning@btinternet.com (L.M.B.); j.m.gambell@gmail.com (J.M.G.); susan.jebb@phc.ox.ac.uk (S.A.J.); 2 Human Development and Health Academic Unit, Faculty of Medicine, University of Southampton, Tremona Road, Southampton SO16 6YD, UK; E-Mails: A.West@soton.ac.uk (A.L.W.); jm24@soton.ac.uk (J.M.); 3 Nuffield Department of Primary Care Health Sciences, University of Oxford, Woodstock Road, Oxford OX2 6GG, UK; 4 NIHR Southampton Biomedical Research Centre, University of Southampton and University Hospital Southampton NHS Foundation Trust, Tremona Road, Southampton SO16 6YD, UK; 5 Department of Biological Sciences, Faculty of Science, King Abdulaziz University, Jeddah 21589, Kingdom of Saudi Arabia

**Keywords:** EPA and DHA supplementation, n-3 fatty acid, n-6 fatty acid, fatty acid displacement, plasma fatty acid fractions, blood cell fatty acids

## Abstract

Eicosapentaenoic acid (EPA) and docosahexaenoic acid (DHA) are increased in plasma lipids and blood cell membranes in response to supplementation. Whilst arachidonic acid (AA) is correspondingly decreased, the effect on other fatty acids (FA) is less well described and there may be site-specific differences. In response to 12 months EPA + DHA supplementation in doses equivalent to 0–4 portions of oily fish/week (1 portion: 3.27 g EPA+DHA) multinomial regression analysis was used to identify important FA changes for plasma phosphatidylcholine (PC), cholesteryl ester (CE) and triglyceride (TAG) and for blood mononuclear cells (MNC), red blood cells (RBC) and platelets (PLAT). Dose-dependent increases in EPA + DHA were matched by decreases in several n-6 polyunsaturated fatty acids (PUFA) in PC, CE, RBC and PLAT, but were predominantly compensated for by oleic acid in TAG. Changes were observed for all FA classes in MNC. Consequently the n-6:n-3 PUFA ratio was reduced in a dose-dependent manner in all pools after 12 months (37%–64% of placebo in the four portions group). We conclude that the profile of the FA decreased in exchange for the increase in EPA + DHA following supplementation differs by FA pool with implications for understanding the impact of n-3 PUFA on blood lipid and blood cell biology.

## 1. Introduction

A diet rich in oily fish containing high concentrations of the long-chain omega-3 (n-3) polyunsaturated fatty acids (PUFA) eicosapentaenoic acid (EPA; 20:5n-3) and docosahexaenoic acid (DHA; 22:6n-3) has been associated with health benefits, particularly a reduced risk of cardiovascular disease [1,2]. Studies with EPA and DHA in supplemental form report a wide range of effects on cardiovascular risk factors amongst other outcomes [2,3] that may explain the benefits of oily fish. EPA and DHA have been used clinically as an adjuvant therapy to prevent secondary myocardial infarction [4], to lower plasma triglycerides [5] and to prevent or alleviate inflammatory conditions including asthma, eczema and rheumatoid arthritis [6] with varying degrees of success [1,2,3]. Whilst not fully elucidated, the mechanisms underlying the health benefits of EPA and DHA, and of oily fish, have been partially attributed to EPA and DHA displacing other fatty acids (FA), notably the omega-6 (n-6) PUFA arachidonic acid (AA; 20:4n-6), in phospholipids of cell membranes [7]. Indeed, it has been well established that in response to supplementation with EPA and DHA, these fatty acids are incorporated in increased amounts into plasma phospholipids [8] and cell membranes including those of platelets [9], mononuclear cells [7], red blood cells [8,10] and other cells such as those of the myocardium [11]. This increased content of EPA and DHA in cell membranes alters the physical properties of the membrane such as its fluidity, which can impact on receptor migration and lipid raft formation, and alter cell signalling pathways which in turn influence cell and tissue responses linked to metabolism, hormone sensitivity, immune function and so on [12]. Furthermore, a decrease in cell membrane AA content and an increase in EPA and DHA alters the balance of eicosanoid and cytokine production from a generally pro-inflammatory profile to a less inflammatory and even inflammation resolving profile [7]. Because of the opposing actions of AA and of EPA and DHA in inflammation, immunity and blood clotting, there has been considerable focus on the ability of EPA and DHA to decrease the AA content of blood and cellular lipids. This has drawn attention away from effects that EPA and DHA might have on the content of other FA in blood lipids, cells and tissues. Consequently, the effect of increased EPA and DHA intake (and incorporation) on the proportions of FA other than AA in different plasma lipids and blood cells is not well described. It is possible that there are cell-specific differences in the FA that EPA and DHA replace which has implications for the metabolic and functional effects of EPA + DHA supplementation.

We previously reported the patterns of increased EPA and DHA incorporation seen in different plasma lipids and blood cells when individuals increased their intake of those fatty acids over the course of 12 months [13]. We now report the patterns of change in other FA observed in this study; we report findings for plasma phosphatidylcholine (PC), cholesteryl esters (CE) and triglycerides (TAG) and for blood mononuclear cells (MNC), red blood cells (RBC) and platelets (PLAT).

## 2. Experimental Section

### 2.1. Original Trial

Data used for this analysis were from a two-centre study examining changes in FA profiles of various blood and tissue fractions in response to 12 months supplementation equivalent to the amounts of marine n-3 PUFA provided by 0, 1, 2 and 4 portions of oily fish per week (one portion = 1.5 g EPA + 1.77 g DHA) [13]. Placebo capsules of high oleic sunflower oil balanced the intake of active capsules. The study was registered at www.controlled-trials.com as ISRCTN48398526 and is described in detail elsewhere [13]. All procedures were approved by the Suffolk Local Research Ethics Committee (approval 05/Q0102/181), and written informed consent was obtained from all participants. The study participants were all non-oily fish consumers and were stratified by age and sex [14], had a BMI range of 18.5–34.9 (median = 25.2) kg/m^2^ and were all described as healthy.

Background diet was monitored by participants completing unweighed 4-day diet diaries recording intakes as estimated portions over three weekdays and one weekend day at 0, 6 and 12 months of the intervention period. At the nine study visits over the 12 month study period, participants were also asked specific questions relating to cooking oils and spreads and white fish consumption. Data were analysed using an in-house database [15] and, as reported previously, there were no significant differences between groups or between time points for total reported energy or macronutrient intake [13]. Compliance to the intervention was assessed by return of capsule blister packs and was high (mean: 98.1%; IQR: 2.2) as previously reported [13].

For this analysis, data for blood samples taken at baseline and after 12 months of supplementation were used. The preparation and analysis of samples has been described previously [13]. Briefly, plasma was prepared from fasting blood collected into heparin and the plasma lipid was further separated into the major fractions phosphatidylcholine (PC), cholesteryl esters (CE) and triglycerides (TAG) by solid-phase extraction on aminopropylsilica cartridges. Mononuclear cells (MNC) and red blood cells (RBC) were isolated from heparinised blood and platelets (PLAT) from citrated blood. FA were analysed as FA methyl esters by gas chromatography, performed on a Hewlett Packard 6890 gas chromatograph fitted with a BPX-70 column (30 m × 0.22 mm × 0.25 μm). The instrument was controlled by, and data were collected using, HPChemStation (Hewlett-Packard Co., Amsterdam, The Netherlands). Full details of lipid extraction methodology, FA methyl ester formation, and gas chromatography running conditions may be found elsewhere [13]. FA methyl esters were identified by comparison of retention times with those of authentic standards run previously [13]. FA are expressed as weight percentage of total fatty acids present in the lipid pool.

### 2.2. Data Analysis

Multinomial linear regression analysis was used to identify the important FA associated with the change in EPA and DHA. Median values for change in the proportion of the 37 FA within each pool were ranked. A threshold median change of 0.1% was set for each pool for inclusion in the model as an indication of potential change in proportion of FA in response to the intervention. A higher threshold of 0.2% median change was used for MNC and PLAT as the model was saturated by the inclusion of a large number of FA using a median change cut-off of 0.1%. A correlation matrix was used for each pool to identify highly correlated (*r* > 0.7) FA. In these situations only a single FA was added into the model at a time, in order to prevent colinearity destabilising the model, but both of the FA were considered important in further analyses. A multinomial regression model was built with change in EPA and DHA as the dependent variables and the FA which exceeded the threshold for each lipid pool as predictors. Other variables including age, sex and dietary change were tested as covariates. From this initial model a backward elimination procedure was used to find a more parsimonious model in order to identify the FA most influential in the change in EPA and DHA in each lipid pool.

In order to visualise patterns in FA classes which are important in each FA pool and the relative magnitude of FA changes, linear combinations were calculated of the coefficients of associations of each FA with EPA and with DHA for all the FA identified in each pool. The combined coefficient represents the change in EPA + DHA for a one unit increase in FA; therefore a FA which has a small magnitude of change has a large coefficient. In order to visualise the relative magnitude of FA changes the reciprocal of the combined coefficient was calculated and compared for each pool.

To determine whether the identified FA were reduced in a dose-dependent manner according to the EPA + DHA supplementation, the effect of dose on the change in each FA was assessed by linear regression analysis. Age and sex, and change in dietary fats (total SFA, MUFA, n-6 PUFA or n-3 PUFA) were tested as covariates in each model and retained if they had an effect.

The effect of the dose on the change in total n-6:n-3 PUFA ratio from baseline to 12 months in each of the pools was determined by mixed effect models adjusted for age and sex. The overall effect of dose over the 12 month visit was tested by a chi test (3 df) contrast of marginal linear predictions from mixed models for each pool.

All data were analysed with Stata version 13 (StataCorp, TX, USA). In all cases a value of *p* < 0.05 was taken to indicate statistical significance.

## 3. Results

### 3.1. Prevalence of FA in the Different Pools at Baseline

The relative proportions of FA in the different pools at baseline are shown in Table 1. The five most prevalent FA in each pool (palmitic acid (PA; 16:0), stearic acid (SA; 18:0), oleic acid (OA; 18:1n-9), linoleic acid (LA; 18:2n-6), and AA) are consistent between blood cells (MNC, PLAT, RBC). These five most abundant fatty acids make up 74%–91% of the total FA in each pool but differ in proportions between pools. PA, OA and LA, which are the most abundant FA in the average Western diet, are strongly represented in every lipid pool examined. AA is one of the most abundant FA in the blood cells and in plasma PC and CE. The n-6:n-3 PUFA ratio was high in plasma CE (median 37.3), low in RBC (5.44) but comparable in all other pools (MNC: 12.1; PLAT: 13.8; plasma PC: 15.0; plasma TAG 12.0). The AA:EPA + DHA ratio was higher in MNC (median 5.93) than in other pools (PLAT: 3.23; RBC: 2.22; plasma PC: 2.05; plasma CE: 4.09; plasma TAG: 1.65).

**Table 1:** Relative abundance of fatty acid (FA) in each lipid pool at baseline.

Fatty Acid	MNC	PLAT	RBC	CE	PC	TAG
**10:0**	0.14 (0.25)	0.03 (0.05)	0.00 (0.07)	0.06 (0.04)	0.00 (0.00)	0.00 (0.00)
**12:0**	0.05 (0.16)	0.08 (0.08)	0.00 (0.00)	0.06 (0.04)	0.00 (0.00)	0.06 (0.15)
**13:0**	0.03 (0.11)	0.00 (0.00)	0.00 (0.00)	0.13 (0.14)	0.00 (0.00)	0.00 (0.13)
**14:0**	0.41 (0.35)	0.74 (0.40)	0.25 (0.12)	0.49 (0.38)	0.27 (0.14)	1.49 (1.09)
**14:1n-9**	0.16 (0.21)	0.06 (0.09)	0.00 (0.00)	0.04 (0.02)	0.00 (0.00)	0.08 (0.10)
**15:0**	0.19 (0.14)	0.18 (0.07)	0.09 (0.14)	0.15 (0.07)	0.15 (0.21)	0.27 (0.08)
**15:1**	0.14 (0.36)	0.07 (0.21)	2.21 (1.99)	0.05 (0.03)	0.05 (0.03)	0.05 (0.04)
**16:0**	**18.2 (2.94)**	**20.7 (2.54)**	**19.3 (1.83)**	**11.3 (1.14)**	**28.9 (1.80)**	**25.6 (3.89)**
**16:1n-7**	1.13 (1.44)	1.97 (1.14)	0.38 (0.19)	**2.80 (1.74)**	0.70 (0.35)	**3.49 (1.50)**
**17:0**	0.32 (0.20)	0.26 (0.06)	0.27 (0.08)	0.07 (0.08)	0.35 (0.10)	0.30 (0.13)
**17:1n-8**	0.30 (0.37)	0.91 (0.90)	4.05 (0.63)	0.05 (0.05)	0.19 (0.11)	0.19 (0.10)
**18:0**	**17.4 (4.95)**	**8.67 (3.07)**	**16.2 (1.27)**	0.76 (0.25)	**12.8 (1.39)**	**3.07 (1.02)**
**18:1n-9t**	1.94 (1.33)	0.17 (0.07)	0.16 (0.22)	1.31 (0.43)	1.55 (0.32)	2.44 (0.62)
**18:1n-9c**	**16.2 (2.75)**	**19.9 (3.55)**	**13.1 (1.45)**	**19.1 (2.66)**	**11.2 (1.69)**	**39.8 (3.96)**
**18:2n-6t**	0.00 (0.19)	0.14 (0.12)	0.21 (0.26)	0.00 (0.00)	0.00 (0.00)	0.00 (0.00)
**18:2n-6c**	**11.4 (9.04)**	**25.1 (7.42)**	**9.93 (1.94)**	**51.7 (6.42)**	**22.4 (3.76)**	**16.6 (5.63)**
**18:3n-6**	0.20 (0.33)	0.43 (0.25)	0.09 (0.16)	0.95 (0.58)	0.11 (0.08)	0.34 (0.23)
**18:3n-3**	0.22 (0.30)	0.62 (0.31)	0.32 (0.33)	0.56 (0.25)	0.22 (0.15)	1.00 (0.49)
**20:0**	0.53 (0.62)	0.26 (0.19)	0.10 (0.16)	0.13 (0.05)	0.15 (0.07)	0.28 (0.16)
**20:1n-9**	0.60 (1.00)	0.35 (0.33)	0.36 (0.22)	0.06 (0.09)	0.20 (0.10)	0.29 (0.14)
**20:2n-6**	0.46 (0.60)	0.20 (0.07)	0.22 (0.18)	0.07 (0.05)	0.34 (0.11)	0.15 (0.09)
**20:3n-3**	0.04 (0.19)	0.10 (0.06)	0.15 (0.27)	0.00 (0.00)	0.00 (0.22)	0.00 (0.00)
**20:3n-6**	1.88 (1.42)	1.63 (0.50)	1.75 (0.56)	0.76 (0.26)	3.30 (1.11)	0.29 (0.14)
**20:4n-6**	**16.0 (9.19)**	**9.54 (4.18)**	**15.5 (2.14)**	**6.59 (2.10)**	**9.55 (2.39)**	1.55 (0.82)
**20:4n-4**	0.22 (0.50)	0.00 (0.00)	0.00 (0.00)	0.08 (0.04)	0.18 (0.11)	0.07 (0.11)
**20:5n-3**	0.57 (0.46)	1.00 (0.52)	1.42 (1.12)	0.91 (0.64)	1.05 (0.57)	0.22 (0.41)
**21:0**	0.30 (0.50)	0.13 (0.17)	0.37 (0.46)	0.03 (0.04)	0.06 (0.09)	0.00 (0.00)
**22:0**	0.18 (0.32)	0.23 (0.10)	0.20 (0.24)	0.00 (0.03)	0.03 (0.14)	0.00 (0.00)
**22:2n-6**	0.07 (0.21)	0.00 (0.00)	0.00 (0.00)	0.00 (0.00)	0.00 (0.00)	0.00 (0.08)
**22:4n-6**	0.76 (1.00)	0.46 (0.40)	2.80 (0.89)	0.03 (0.04)	0.34 (0.19)	0.14 (0.07)
**22:5n-3**	1.48 (0.84)	0.88 (0.26)	3.11 (0.60)	0.07 (0.08)	0.95 (0.28)	0.30 (0.43)
**22:5n-6**	0.37 (0.74)	0.13 (0.05)	0.47 (0.23)	0.04 (0.04)	0.23 (0.18)	0.17 (0.22)
**22:6n-3**	1.86 (0.76)	1.97 (0.71)	5.34 (1.88)	0.63 (0.34)	3.47 (1.51)	0.72 (0.46)
**23:0**	0.04 (0.17)	0.05 (0.07)	0.00 (0.00)	0.00 (0.00)	0.00 (0.00)	0.00 (0.26)
**24:0**	0.60 (1.16)	0.10 (0.05)	0.38 (0.13)	0.00 (0.00)	0.00 (0.00)	0.00 (0.00)
**24:1n-9**	0.20 (0.28)	0.00 (0.00)	0.00 (0.00)	0.04 (0.04)	0.25 (0.14)	0.00 (0.13)

Data are median (IQR). The five most prevalent FA in each pool are indicated in bold. MNC (mononuclear cells); PLAT (platelets); RBC (red blood cells); CE (plasma cholesteryl esters); PC (plasma phosphatidylcholine); TAG (plasma triglycerides).

### 3.2. Change in FA Profile in Each Lipid Pool

**Figure 1:**
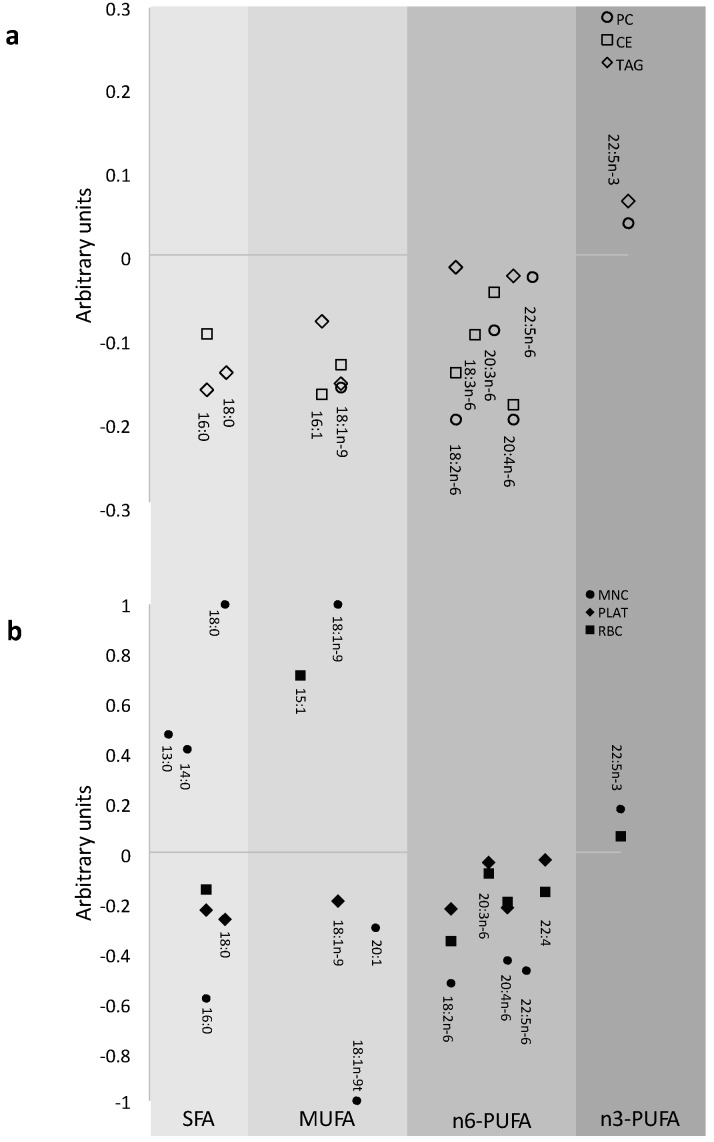
Patterns of modelled changes in fatty acids which occur with the increase in eicosapentaenoic acid (EPA) and docosahexaenoic acid (DHA) in (**a**) plasma and (**b**) blood cell fatty acid pools. Linear combinations of the co-efficients from the multinomial linear regression models were calculated for each fatty acid important for the change in EPA + DHA. As a small regression coefficient for the fatty acid of interest reflected a large change in the outcomes EPA + DHA and vice versa the data are the reciprocal of the co-efficients in order to portray the magnitude of effect, and this is presented in arbitrary units. Fatty acids are grouped according to class to depict the patterns of change in fatty acids for each pool.

The FA identified by the multinomial models to be important for change in EPA and DHA are shown for plasma lipid pools in Figure 1a and for blood cells in Figure 1b. The FA displaced in PC were predominantly n-6 PUFA, whereas in TAG they were predominantly MUFA and SFA, and in CE SFA, MUFA and n-6 PUFA were all displaced following EPA and DHA supplementation. In RBC the FA predominantly decreased were n-6 PUFA, in PLAT predominantly SFA and n-6 PUFA, while in MNC there were a range of changes in FA from all classes. Docosapentaenoic acid (DPA; 22:5n-3) increased in most pools (plasma PC and TAG, RBC and MNC) with increased EPA and DHA intake.

### 3.3. Effect of Dose of EPA and DHA on the Change in Identified FA in Each Pool

**Figure 2:**
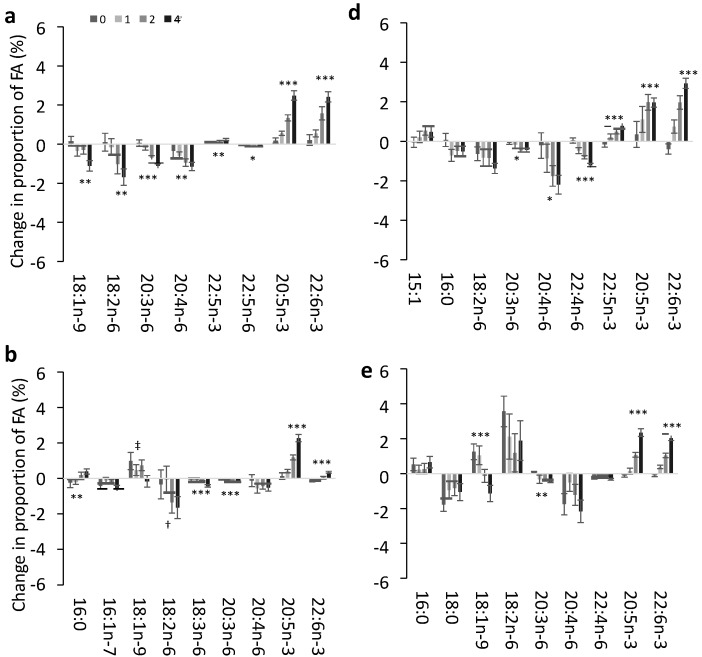

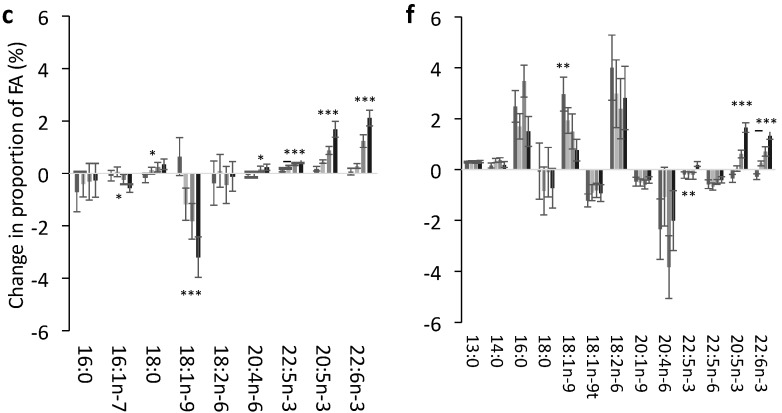
Mean change in key fatty acids in response to 12 months supplementation of EPA + DHA equivalent to 0, 1, 2 or 4 portions of fish per week in different plasma and blood cell pools. The observed mean ± SE change from baseline in the fatty acids identified as important in relation to change in EPA + DHA in the multinomial regression models in (**a**) Plasma PC; (**b**) Plasma CE; (**c**) Plasma TAG; (**d**) RBC; (**e**) PLAT; (**f**) MNC. The effect of dose was tested by linear regression models for each fatty acid. Each model was tested with and without age and sex which were included as covariates if significant. Change in dietary SFA, MUFA, n-3 PUFA or n-6 PUFA where relevant were also tested to determine if change in diet influenced the change in fatty acids with EPA + DHA dose. Significant effects of dose detected in these models are shown as: * *p* < 0.05; ** *p* < 0.01; *** *p* < 0.0001; ^†^ Effect of dose (*p* = 0.05) only when taking into account the change in dietary n-6 PUFA from baseline to 12 months; ^‡^ Effect of dose (*p* < 0.05) is no longer significant when taking into account the effect of change in dietary MUFA.

The effect of the dose of EPA + DHA supplementation on the change in FA identified by the multinomial models is shown for each lipid pool in Figure 2. In plasma PC (Figure 2a) there was a clear dose response such that OA and three n-6 PUFAs (LA, di-homo-γ-linolenic acid (DGLA; 20:3n-6 and AA) were decreased in relation to an increasing dose (and increasing incorporation) of EPA + DHA. In plasma CE (Figure 2b) the n-6 PUFAs LA, γ-linolenic acid (GLA; 18:3n-6) and DGLA (when taking into account the change in dietary n-6 PUFA) decreased in a dose-dependent manner with increasing EPA + DHA. There was a dose-dependent decrease in OA, but this was not seen when changes in dietary MUFA intake were accounted for. PA was also increased in a dose-dependent manner in CE. In plasma TAG (Figure 2c) the only dose-dependent decreases were in MUFA (palmitoleic acid (POA; 16:1n-7) and OA). In RBC there was a significant dose-response decrease in the n-6 PUFAs DGLA, AA and docosatetraenoic acid (DTA; 22:4) (Figure 2d). In PLAT DGLA was decreased and there was also a trend (*p* = 0.09) for DTA to be decreased in a dose-dependent manner (Figure 2e). In both PLAT and MNC OA increased, but the magnitude of the increase was inversely proportional to dose. There was also a pattern for LA to be increased in MNC and PLAT in an inverse dose-responsive manner, although this was not significant (Figure 2d,e). DPA was increased in a dose-dependent manner in the four pools in which it was identified as an important contributor to change in EPA and DHA.

### 3.4. Impact of EPA and DHA Supplementation on the FA Profile

Although EPA and DHA increased in all pools, the most abundant FA remained the same as at baseline, even though the proportions of these FA were often changed. The only exception was in plasma CE, where EPA displaced POA at the highest intake of EPA + DHA as one of the five most abundant FA (at 12 months, EPA mean: 3.49, SD: 1.03; POA mean: 2.81, SD: 1.20).

The increase in EPA, DPA and DHA and the decrease in n-6 PUFAs resulted in a significant decrease in the n-6:n-3 PUFA ratio in each of the FA pools (Table 2). The ratio was decreased in the four portions group to 37%–64% compared to the placebo group across the FA pools (Table 2).

The AA:EPA + DHA ratio was also lower in all pools, such that after 12 months of the four portions dose the median values were MNC: 2.53; PLAT: 1.11; RBC: 1.13; plasma PC: 0.81; plasma CE: 1.21; plasma TAG: 0.40.

**Table 2:** The n-6:n-3 PUFA ratio in the 0 portions group and differences by dose in the change in the ratio after 12 months of EPA + DHA supplementation.

	n-6:n-3 PUFA Ratio	Change in n-6:n 3 PUFA Ratio ^‡^			Overall Effect of Dose at 12 Months (*p*)
	0 Portion Value ^†^	1 Portion	2 Portion	4 Portions	
Plasma PC	14.4 ± 0.59	−3.32	−5.41	−7.48	<0.0001
		(−4.90, −1.73)	(−7.06, −3.77)	(−9.09, −5.87)	
Plasma CE	35.5 ± 1.50	−7.54	−14.5	−20.7	<0.0001
		(−11.5, −3.51)	(−18.7, −10.4)	(−24.8, −16.6)	
Plasma TAG	10.0 ± 0.49	−1.35	−2.88	−4.59	<0.0001
		(−2.65, −0.05)	(−4.23, −1.54)	(−5.90, −3.27)	
RBC	4.90 ± 0.27	−0.82	−1.31	−1.81	<0.0001
		(−1.54, −1.04)	(−2.06, −0.55)	(−2.54, −1.07)	
PLAT	15.3 ± 0.70	−4.17	−6.56	−9.76	<0.0001
		(−5.69, −2.64)	(−8.13, −4.99)	(−11.3, −8.22)	
MNC	16.2 ± 0.69	−3.20	−6.24	−8.71	<0.0001
		(−5.04, −1.36)	(−8.15, −4.33)	(−10.6, −6.85)	

Mixed-effects models for the change in n-6:n-3 PUFA ratio in each pool adjusted for age and sex. The overall effect of dose over the 12 month visit by 3df chi test contrast of marginal linear predictions from mixed model is presented for each pool. **^†^** 0 portion values are mean ± standard error; **^‡^** Adjusted mean differences (95% CI) between groups at 12 months calculated in the mixed effects models.

## 4. Discussion

In this study we show that the dose dependent incorporation of EPA and DHA following 12 months supplementation is matched by dose dependent decreases in other FA in all pools, but that the FA that change differ between pools. For the plasma fractions PC and CE, EPA and DHA predominantly displaced a range of n-6 PUFAs, whereas it was predominantly the MUFA OA which was displaced in TAG. In blood cells EPA and DHA displaced a range of n-6 PUFA, including but not limited to AA in RBC and PLAT, whereas in MNC a number of FA changes occurred across all classes of FA.

Decreases in n-6 PUFA were the most important compensatory FA changes in response to the increase in EPA and DHA in many pools and this was reflected in a change in total n-6:n-3 PUFA ratio in all pools. An increase in EPA and DHA intake equivalent to a dietary change of four portions of oily fish per week will reduce the n-6:n-3 PUFA ratio by around half in all of the FA pools. This effect on the n-6:n-3 PUFA ratio has been reported consistently from long-term (12 month) high dose (~10 g/day) n-3 PUFA supplementation [16] to comparatively low dose (0.8 g/day) n-3 PUFA for a comparatively short supplementation period of 10 weeks [17].

AA decreased in most pools, but significant dose-dependent decreases in AA were only observed in plasma PC and in RBC, although prominent non-dose dependent decreases were also observed in PLAT and MNC. There has been a longstanding interest in compensatory decreases in AA following EPA and DHA supplementation, due to the role of AA as a primary precursor of eicosanoids [7]. AA is one of the five most abundant FA in most of the pools, whereas significant dose-dependent changes of a comparable magnitude also were observed for DGLA in plasma PC and CE, RBC and PLAT where it was 3–10 fold less abundant, meaning these changes may have more biological impact. DGLA is itself important for eicosanoid synthesis and regulation of pathways involving other bioactive lipid compounds [7,18]. A significant decrease in DGLA but not AA was previously reported following supplementation with 3 g/day long chain n-3 PUFA for 10 weeks [17]. This was accompanied by a decrease in AA-derived pro-inflammatory mediators in plasma [17]. Likewise changes in other low abundance n-6 PUFA (Adrenic acid (AdA; 22:4n-6) in RBC and PLAT, and GLA in plasma CE) may be of biological importance. Changes to these other n-6 PUFA in response to EPA and DHA supplementation are not widely reported in the literature.

The AA:EPA + DHA ratio was higher in MNC than other blood cell types. This ratio may reflect the importance of AA as a precursor for eicosanoids involved as mediators and regulators of inflammation and the immune response [7]. Although AA:EPA + DHA was decreased in all lipid pools with increasing EPA + DHA intake, the decrease may have especially pronounced effects on the function of MNC and PLAT, where AA serves important roles as an eicosanoid precursor. The changes in the ratio of AA:EPA + DHA observed with increased consumption of EPA + DHA are strongly linked to the impact of the latter on inflammation [7] and thrombosis [2,3].

In addition to compensatory decreases in n-6 PUFA seen in most pools, prominent changes in MUFA and SFA were also observed in plasma TAG, plasma CE, MNC and PLAT. A FA profile change from MUFA or particularly SFA to n-3 PUFA is likely to have structural effects on membranes altering fluidity [19] and lipid raft formation with subsequent effects on membrane receptor function and cell signalling [12]. Thus these changes in FA class are also of interest, particularly in PLAT and MNC. Changes in FA class of membranes have not been consistently shown in response to EPA and DHA supplementation as summarised by Hodson *et al.* [20] although many previous studies tended to be of shorter duration (≤12 weeks).

A pattern was evident in PLAT and MNC for OA and LA and in plasma CE for OA to be increased in an inverse dose-dependent manner (although this was not significant for LA), such that the increase was lowest for the highest dose of EPA and DHA. This is most likely explained by OA and LA provided by the placebo capsules, where the dose provided was inversely proportional to EPA and DHA (0 portions: 10.41 g OA + 1.92 g LA per week; four portions: 24.3 g OA + 4.48 g LA per week). A wide range of changes including increases in a number of SFA were noted in MNC. The incorporation of n-3 PUFA into the MNC membranes may have stimulated a number of FA changes in the membranes of these cells in order to maintain a constant membrane fluidity. It has previously been suggested that increased incorporation of n-3 PUFA into phospholipid membranes triggered changes in phospholipid class in cell membranes in order to maintain constant membrane fluidity [21]. There were clearly different patterns of FA displacement with the different pools studied here. In addition to a potential impact on membrane fluidity, altered FA composition of cell membranes can influence formation of lipid rafts and the activity of various types of membrane proteins and can alter signalling pathways that ultimately control cell and tissue responses linked to metabolism, hormone sensitivity, lipid mediator production, and function [12]. Thus, the FA changes described here are likely to be of functional relevance.

As this was a placebo-controlled trial, the potential impact of the high oleic sunflower oil placebo capsules (see Browning *et al.* [13] for full composition details) must be considered in the interpretation of the FA changes. Whilst this placebo was chosen to have minimal impact on FA composition of target lipid pools by closely mimicking the habitual diet, the inverse dose-response increase in OA (and trend in LA) noted in some pools indicate a potential impact. It is possible that changes in LA may also impact on changes in other n-6 PUFA. Dietary changes in FA would also contribute to changes in FA profiles and we attempted to account for this in our analyses. Although dietary data were collected at the start and the end of the 12 month study, these data only included total FA classes rather than individual FA contributions. Furthermore, this dietary information was captured by a four day food diary, which is not a robust indicator of habitual food intake. Whilst these measures, plus an additional assessment of diet conducted halfway through the trial, indicated no substantial changes in dietary habits at these three discrete time points [13], small changes in dietary fat intake in the intervening periods would none-the-less have an impact on the FA profile.

## 5. Conclusions

The profile of the FA decreased in compensation for the increase in EPA and DHA following supplementation differs by FA pool, and this may have important biological implications beyond the previously reported decreases in AA.
